# Plastids of Marine Phytoplankton Produce Bioactive Pigments and Lipids

**DOI:** 10.3390/md11093425

**Published:** 2013-09-09

**Authors:** Parisa Heydarizadeh, Isabelle Poirier, Damien Loizeau, Lionel Ulmann, Virginie Mimouni, Benoît Schoefs, Martine Bertrand

**Affiliations:** 1 MicroMar, Mer Molécules Santé/Sea Molecules & Health, IUML-FR 3473 CNRS, LUNAM, University of Le Mans, Avenue Olivier Messiaen, Le Mans 72000, France; E-Mails: parisa_ht@yahoo.com (P.H.); schoefs@univ-lemans.fr (B.S.); 2 Microorganisms, Metals and Toxicity, Cnam SITI CASER STM, BP 324, Cherbourg Cedex 50103, France; E-Mails: isabelle.poirier@cnam.fr (I.P.); damien.loizeau@cnam.fr (D.L.); 3 MicroMar, Mer Molécules Santé/Sea Molecules & Health, IUML-FR 3473 CNRS, LUNAM, University of Le Mans, IUT Génie Biologique, Laval Cedex 53020, France; E-Mails: lionel.ulmann@univ-lemans.fr (L.U.); virginie.mimouni@univ-lemans.fr (V.M.)

**Keywords:** plastids, carotenoids, cyanobacteria, microalgae, polyunsaturated fatty acids, tetrapyrroles

## Abstract

Phytoplankton is acknowledged to be a very diverse source of bioactive molecules. These compounds play physiological roles that allow cells to deal with changes of the environmental constrains. For example, the diversity of light harvesting pigments allows efficient photosynthesis at different depths in the seawater column. Identically, lipid composition of cell membranes can vary according to environmental factors. This, together with the heterogenous evolutionary origin of taxa, makes the chemical diversity of phytoplankton compounds much larger than in terrestrial plants. This contribution is dedicated to pigments and lipids synthesized within or from plastids/photosynthetic membranes. It starts with a short review of cyanobacteria and microalgae phylogeny. Then the bioactivity of pigments and lipids (anti-oxidant, anti-inflammatory, anti-mutagenic, anti-cancer, anti-obesity, anti-allergic activities, and cardio- neuro-, hepato- and photoprotective effects), alone or in combination, is detailed. To increase the cellular production of bioactive compounds, specific culture conditions may be applied (e.g., high light intensity, nitrogen starvation). Regardless of the progress made in blue biotechnologies, the production of bioactive compounds is still limited. However, some examples of large scale production are given, and perspectives are suggested in the final section.

## 1. Introduction

Phytoplankton was coined in 1897 using the Greek words φυτόν (*phyton*), meaning “plant”, and πλαγκτός (*planktos*), meaning “wanderer” or “drifter” [1]. Phytoplankton is therefore a generic term designing unicellular prokaryotic and eukaryotic organisms. Most of the taxa are autotrophic but taxa such as *Polytoma* sp., *Polytomella* sp., *Prototheca* *wickerhamii*, *etc.*, have been described with degenerated chloroplasts [2,3]. These taxa grow heterotrophically in the dark whereas other algae ordinarily grow phototrophically in the light. Despite the fact that the number of newly described species is increasing yearly [4,5,6] only a small percentage of species belonging to phytoplankton have been described so far [7]. Phytoplankton has colonized every type of ecological niche, and it constitutes the dominant group of living organisms in term of species diversity in marine waters [8].

Phytoplankton was playing crucial roles during Earth evolution. For instance, cyanobacteria were at the origin of the rise of the atmospheric oxygen, an event known as the Great Oxidation Event (GOE) [9,10,11]. Today, the contribution of phytoplankton to the biosphere continues to be unique because this group largely contributes to the renewal of the atmospheric oxygen and acts as a tremendous sink for CO_2_, which is used for the synthesis of organic compounds through photosynthesis [12]. This function makes phytoplankton crucial primary producers on which every food chain relies. While accounting for less than 1% of Earth’s biomass, phytoplankton is responsible for more than 50% annual net biomass production [13,14]. It is known for ages that marine organisms, including phytoplankton, constitute a pretty diversified source of compounds interesting for health, well-being, cosmetics, new fuels *etc*. [7,15,16,17,18]. Actually, it is estimated that approximately 20,000 active substances originating from marine organisms are used in the industry and can be used as an alternative source of chemically synthesized medicine [19]. As mentioned earlier, phytoplankton differentiates from other planktonic taxa by the presence of photosynthetic membranes. In prokaryotic species, the photosynthetic membranes are located within the cytosol whereas in eukaryotic species, they are enclosed in a cell compartment denoted as the chloroplast (for a review, see [20]). Beside the photosynthetic activity, chloroplasts are involved in many biosynthetic pathways and constitute as much as real cellular factories synthesizing crucial cell components such as lipids and pigments [21].

This manuscript starts with reminders on plastid phyllogeny. Then two families of compounds are considered, *i.e.*, pigments and lipids; their diversity is described and their health benefits are detailled. Tetrapyrroles and carotenoids have strong anti-oxidant, anti-inflammatory and anti-mutagenetic activities, whereas lipids (essentially polyunsaturated fatty acids) have anti-cholesterol and cardioprotective effects. The following section deals with the cellular increased production of the bioactive molecules by variation of environmental parameters. Because plastids derived from photosynthetic prokaryotes, cyanobacteria have been also considered in this review.

## 2. Plastid Phylogeny

According to the fossil record cyanobacteria were already present over 2 billion years ago [22]. Based on the assumption that cyanobacteria were responsible of the GOE, it is thought that this phylum existed already 2.45–2.32 Bya [9,10,11]. The impact of GOE cannot be emphasized enough as this event changed Earth’s history by enabling the evolution of aerobic life [23]. Oxygen accumulation in the atmosphere was the result of oxygenic photosynthesis. This process required four macrocomplexes, namely the water-oxidizing photosystem II (PSII), cytochrome b_6_/f, photosystem I (PSI), and the H^+^-translocating ATP synthetase (CF_0_F_1_) [24]. They supply ATP and NADPH for synthesis of many essential compounds for autotrophic growth and the synthesis of many compounds including those discussed in this contribution.

A photosystem is a pigment-protein complex composed of a reaction center (RC) and a light-harvesting complex (LHC). Two families of pigments are found in LHCs: the tetrapyrroles and carotenoids [25,26]. Besides the typical closed tetrapyrrole chlorophyll (Chl) *a* and β-carotene, cyanobacteria harbour usually two types of accessory pigments, *i.e.*, open tetrapyrroles (phycobilins) and glycosylated xanthophylls [27]. Phycobilins (PBS) are also accessory pigments in red algae and Glaucophytes. In the other eukaryotic phytoplanktonic taxa, Chla *a* is accompanied by another type of Chl (Table 1). Chlorophyllin (Chlin) is a water-soluble semi-synthetic derivative of Chl that is widely used as a food colorant, and is commercially available as an herbal “remedy”. Therefore, although not natural, this compound was also considered in this review.

On the basis of molecular, biochemical and cellular arguments, it is classically assumed that eukaryotic phytoplankton derives from the internalization of a cyanobacterium-type organism into a eukaryotic heterotrophic cell, the so-called first endosymbiotic act (for a review, see [28]). The reconstitution of true phylogenetic history of organisms has become a very important issue in biology. The availability of numerous molecular sequences allowed biologists to propose new hypotheses for evolutionary histories of taxa that lead eventually to unresolved or conflicting interpretations and the real history seems more complicated than thought initially (see for instance [29,30,31]) and will not be discussed here. This primary endosymbiotic act explains why today’s chloroplasts have two envelopes (for a review on the photosynthetic membranes in phytoplankton, see [20]). To explain the presence of additional membranes around the chloroplasts, additional endosymbiotic acts are invoked [32], for a review, see [33]. Actually, the recent phylogenetic studies carried out indicated that the history of secondary endosymbionts is more complicated than originally thought because a detailed study of the diatom genes support a putative green algal origin of several genes [14,20,30,31,32,33,34]. The members of the red lineage of secondary endosymbionts constitute a most diverse group of organisms. In these organisms, the gene mixing that occurred along evolution allowed a tremendous diversification of the metabolism and the most important from the pharmaceutical point of view being the diatoms (Heterokonta) and the dinoflagellates (Alveolata).

**Table 1:** Main chlorophyll and carotenoid types in the various taxa of photosynthetic organisms. “+” and “-” mean that the pigment was detected or not in the taxa.

Pigment type	Cyanobacteria	Glaucophytes	Red algae	Brown algae	Diatoms	Green algae	Land plants
PBS	+	+	+	-	-	-	-
Chl *a*	+, except in *Acaryochloris marina* and related taxa	+	+	+	+	+	+
Chl b	except in Prochlorophytes	-	-	-	-	+	+
Chl c	-	-	-	+	+	-	-
Chl d	Only in *Acaryochloris marina* and related taxa	-	-	-	-	-	-
Chl f	Only in filamentous cyanobacteria from stromatolites	-	-	-	-	-	-
β-Carotene	+	+	Unicellular	+	+	+	+
Fucoxanthin	-	-	-	+	+	-	-
Diadinoxanthin	-	-	-	Traces	+	-	-
Diatoxanthin	-	-	-	Traces	+	-	-
Violaxanthin	-	-	+	+	+	+	+
Lutein	Depends on species		Macrophytes	-	-	+	+
Zeaxanthin	Depends on species	+	+	+	Traces	+	+
Xanthophyll cycle	-	-	-	+	+	+	+
Echinenone	Depends on species	-	Traces	Traces	-		*Adonis* sp.
Myxoxanthophyll	Depends on species	-	-	-	-	-	-
Canthaxanthin	*Anabaena*	-	-	-	-	Depen on species	*Adonis* sp.
Aphanizophyll	Depends on species	-	-	-	-	-	-
β-Cryptoxanthin	*Aphanizomenon*	-	+	-	-	+	+
Lycopene	*Nostoc*	-	-	-	-	Depend on species	+
Synechoxanthin	*Synechococcus*	-	-	-	-	-	-

## 3. Bioactivity of Pigments and Lipids from Plastids of Marine Phytoplankton

As mentioned earlier, most of phytoplanktonic species are photoautotrophs. Consequently, they are rich in pigments. Three major classes of photosynthetic pigments are recognized, *i.e.*, (1) Chls and Chl-related molecules, (2) Cars (carotenes and xanthophylls) and (3) PBS. Altogether the pigment family represents several hundreds of purified molecules [25,35]. All these molecules share the unique property to absorb light thanks to their characteristic conjugated double bond network [25,36,37]. Considering the high structural diversity of pigments and the possibility to chemically modify these molecules to obtain better pharmacological properties, the potential of phytoplankton as a source of molecules of therapeutical interest is obviously very high. Unfortunately, the lability and difficult purification of these molecules make the biological activity of most of these molecules unstudied. However, when purified pigments were obtained, they presented a high activity on pharmacological and cellular effectors even at very low concentrations. These molecules can therefore be used for chemoprevention.

The superiority of marine molecules, as compared to analogue terrestrial resources, can be explained by the simultaneous presence of a wider variety of substances in marine organisms. These substances would act synergentically, providing an enhanced bioactivity [38]. Very often, synergistic actions between pigments and lipids are described. For instance, Rao & Rao [39] and Micallef & Garg [40] found a synergistic action between pigments, fatty acids and phytosterols on plasma lipid concentration decrease, on inflammatory response and thus on cardiovascular disease risk prevention.

### 3.1. Pigments

#### 3.1.1. Tetrapyrroles

Human populations regularly have been consuming green vegetables since the discovery of agriculture practices regularly some 15,000 years ago. This traditional and long practice made in the collective unconscious green pigments as save compounds. Indeed, Chl derivatives such as pheophytin have been found in some plants known in traditional medicine to be active against diseases such as *Clinacanthus nutans*-herpes simplex virus [41] and *Lonicar hypoglauca*-hepatitis C virus [42]. Until recently, almost nothing was known about the assimilation of closed tetrapyrroles: it was simply thought that Chl absorption was limited (1%–3%). Actually, the phytol chain of the native Chl molecules would be cleaved whereas the Mg ion is leached upon ingestion, the resulting pheophorbide and pheophytine being excreted in the feces [43]. Major progress in studying tetrapyrrole assimilation was possible when intestinal cell could be grown *in vitro*. Using such a model, *i.e.*, Caco-2 human cells, [44] demonstrated that pheophytin can be absorbed as well. The anti-genotoxicity of natural Chls was revealed by Negishi *et al.* [45] whereas others studies reported the anti-oxidant activity, the modulation of xenobiotic enzyme activity, and the induction of apoptotic event in cancer cells of Chl [46,47]. This anti-cytotoxicity seems specific to natural Chl and could not be demonstrated with non-natural Chl molecules (see below) [48]. Chl precursors, Chl derivatives, or Chl analogs are also used in medicine for photodynamic treatments of cancers, e.g., [49].

The harmlessness of tetrapyrroles, especially Chl does not mean that the ingestion of tetrapyrroles is without danger. For instance, Chl intolerance can occur due to the liberation of the Chl phytol moity in patients suffering from Refsum’s disease. In this pathology, phytanic acid accumulates in serum and other tissues due to a defect of the metabolic pathway for phytanic acid [43]. Lohrey *et al.* [50] demonstrated that albino rats fed with lucerne—but not with ryegrass—protein concentrate, develop photosensitization. This allergy to sunlight could be due to the accumulation of pheophorbide and related pigments in tissues [51]. Similar sunlight allergic reactions were reported in humans having ingested dried laver enriched with pheophorbides and pyropheophorbides [52]. Gandul-Rojas *et al.* [53] have shown that these deesterified tetrapyrroles are more strongly absorbed than the phytylated ones from the food matrix by the intestinal epithelial cells, allowing a larger accumulation in the patient body. Therefore, there is a need for information on the effects of processes on tetrapyrrole bioaccessibility and bioavailability. For instance, conservation industrial processes such as freezing and canning along with cooking have a positive effect on Chl bioavailability [54,55], making the pigment’s quantity after processing exceeding 100% of the value measured before processing (e.g., [56,57]). Non-natural Chls, such as Cu-Chls, Cu-pheophorbide, Cu-pheophytin, or Chlins, are digested similarly to natural Chl molecules, that is, the phytol chain is cleaved but the Cu ion is not leached from the porphyrin ring because the chelate is much more stable. The stability is only relative because an increase in Cu has been measured in the plasma following Chlin ingestion [58]. Several tests suggested that oral or parenteral administration of Chlins did not produce any gross adverse effect on health [59,60].

Phytoplankton contains also other types but usually minor tetrapyrroles such as hemes. Heme-protein complexes are degraded in the lumen of the stomach and small intestine [61]. Hemes being soluble in alkaline environment, they do not need to bind proteins for the luminal absorption, which is indeed facilitated by the presence of specific heme transporters such as the heme carrier protein 1 (HCP1) at the apical surface of enterocytes [62]. Once in the erythrocytes, hemes are cleaved by heme oxygenases yielding to iron, biliverdin and carbone monoxide formation [63]. Iron is driven to a labile iron pool and used for metabolism. Open tetrapyrroles derived from hemes through the oxygenation of the tetrapyrrole ring [64].

##### 3.1.1.1. Anti-Oxidant, Anti-Inflammatory and Anti-Mutagenic Activities

Many diseases such as cancer, diabetes and, neurodegenerative and cardiovascular diseases have in common oxidative stress as a major cause of inflammatory events. The anti-oxidant and anti-inflammatory activities of phytoplanktonic pigments are widely demonstrated and evidenced in numerous *in vitro* free and *in vivo* radical scavenging assays. The presence of a metal-chelate strongly enhances the anti-radical capacity closed tetrapyrroles. Protoporphyrin methyl ester and its magnesium chelated derivative, as well as pheophorbide b and pheophytin b, were also identified as strong anti-oxidant molecules [65]. The ability of the porphyrin ring to transfer electrons explains the anti-oxidant activity of Chls and derivatives. The highest anti-oxidant activity was obtained with of pheophorbide b, compared to pheophorbide *a*, suggesting that the presence of the aldehyde function may be critical to this activity [47]. This was confirmed in a study that investigated the action mechanisms of natural and synthetic porphyrins regarding their anti-inflammatory effects *in vitro* and *in vivo* [66]. Unfortunately, no structure related to the Chl familly of compounds has been tested leaving uncertain the effects of plant pigments on porphyrins targets such as the tumor necrosis factor alpha production, the Fyn tyrosine kinase inhibitors [66]. However, on the basis of the study, some potential for Chl and Chl-derivatives can be hypothesized. More generally, it should be recall that the antioxidant properties of Chls and Chl-derivatives disappear in the presence of light. Actually, the accumulation of free tetrapyrroles in photosynthetic cells leads to a dramatic oxidative stress upon light illumination [67,68,69]. Metal-free and metallo-Chl derivatives have also anti-mutagenic activities, as demonstrated using a bacterial mutagenesis assay [47,70]. Similar properties are exhibited by open-chain tetrapyrroles (anti-oxidative: allophycocyanin [71], phycocyanin [72], phycoerythrin [73] and anti-inflammatory: phycocyanin [74]) *in vitro*. As a conclusion, most microalgal pigments exert strong *in vitro* anti-oxidant activity, but additional intervention trials are required to precise their absorption, metabolism and potential as natural anti-oxidant, anti-inflammatory and anti-mutagenic compounds *in vivo*. Very importantly, the capacity of phycocyanin to quench oxygen radical species is preserved in food supplement [75].

##### 3.1.1.2. Anti-Cancer

Chlin has been reported to have potent anti-mutagenic and anti-carcinogenic effects in short-term tests *in vitro* and *in vivo* [45,46]. There is an abundant literature about the interest of food molecules to preserve health, for reviews see [76]. However, it may happen that food, for instance stored in poor conditions, gets infected by molds such as *Aspergillus flavus* and *A. parasiticus*. These fungi secrete mycotoxin aflatoxin B1 (AFB), one of the most known human carcinogenesis linked to diet. The action mechanism of AFB is pretty well known. However, it is out of the scope of this contribution to present the details of these mechanisms and it is sufficient to known the following facts to understand the interest of tetrapyrroles in thwarting AFB action. Briefly, AFB is metabolized by cytochrome P450 (CYP) enzymes to the exo-AFB-8,9-epoxide (AFBO), which constitutes the main metabolites of the reaction. (for details, see recent reviews, e.g., [77]). AFBO is highly reactive, and can further bind covalently to cellular macromolecules such DNA forming AFB-N7-guanine adducts. These adducts are chemically unstable and rapidly excreted but they are stabilized through rearranging to a ring-opened formamidopyramidine structure. The AFB-DNA-adducts can trigger somatic alterations at transcriptionally active DNA regions when not removed by DNA repair enzymes [78]. The ability of Chlin to form complex with carcinogens is not restricted to AFB (for a review, see [79]) as AFB could also bind heterocyclic amines that favour the development of colorectal adenoma [80]. Heterocyclic amines are for instance produced when meat is cooked at high temperature [80]. Thus, Chlin exhibits the property to chelate AFB yielding to the formation of chemically stable Chlin-AFB complexes, and those complexes are no longer absorbed by intestine and excreted with feces [81]. Moreover, inhibitory effects of Chlin on human CYP have been reported [82]. Based on these effects, Chlin was used recently in a chemointervention trial in China, resulting in a 55% reduction of the urinary AFB-N7-guanine adduct [83]. Shaughnessy *et al.* [84] proposed arguments for a protective action of Chl on DNA damages. The efficiency of dephytylated compounds is less than the phytylated ones [85]. In addition, Chlin has been reported to exhibit an anti-proliferative effect on various cancer cell lines [86,87,88]. Priyadarsini *et al.* [70]) reported that Chlin alterates the expression of genes coding for proteins involved in the transformation of normal to malignant phenotype of buccal pouch cells. However, Nelson [89] reported that commercially available Chlin can have a tumor promoting effect and at present the Chlin content in food is regulated [55].

Microalgae pigments may have interest to restore drug sensitivity or reverse multi-drug resistance (MDR) in cancer cells, as some of them inhibit or down-regulate drug efflux pumps. For instance, a significant reduction of P-glycoprotein expression in drug-resistant hepatocellular carcinoma (R-HepG2) cells, at both transcriptional and translational levels, was observed when cells were treated with pheophorbide *a* [90]. Recently, a natural Chl of the Phaeophyceae, Chl c2, was found to trigger the degranulation of a rat basophilic leukemia cell line (RBL-2H3), whereas Chl *a* and b were unable to do it [91]. In addition, Chl c2 has an anti-allergic power.

##### 3.1.1.3. Miscellaneous

Because of their antioxidant and anti-inflammatory activity, most microalga pigments have neuroprotective effects in cultures rat cerebellar neurons, and hepatoprotective effects in hepatocytes grown *in vitro* (e.g., phycocyanin, phycoerythrin [92]). Besides, some studies have demonstrated anti-viral and anti-fungal activities for some pigments (e.g., allophycocyanin, phycocyanin [92]). The physicochemical characteristics of PBS, Chl and Chl catabolites make them suitable for use as fluorescent probes for cellular and tissular analysis (e.g., cell sorting, cytofluorescence, flow cytometry, histofluorescence, binding assays, reactive oxygen species (ROS) detection, labeling of pathological or apoptotic cells, *etc.*). Phycocyanin- or phycoerythrin-coupled anti-bodies are common reagents available for research and medical use, in which PBS act as powerful and highly sensitive fluorescent probes (for reviews, see [92]).

#### 3.1.2. Carotenoids

Many xanthophylls (violaxanthin, antheraxanthin, zeaxanthin, neoxanthin, lutein, loroxanthin, astaxanthin and canthaxanthin) can be synthesized by microalgae while others (diatoxanthin, diadinoxanthin and fucoxanthin) can also be derived by brown algae ([93]; Table 1). In cyanobacteria carotenoids are much diversified: β-carotene; echinone, synechoxanthin, myxoxanthophyll, canthaxanthin, zeaxanthin, isozeaxanthin, aphanizophyll, lutein, lycopene, β-cryptoxanthin and Orange Carotenoid Protein (OCP) can be found (Table 1). This blue-green light photoactive protein was described very recently; it binds the carotenoid 3′-hydroxyechinenone as its only pigment [94,95]. Cyanobacteria are rich sources of lycopene, and also provide higher ratios of *cis*/all-*trans* lycopene which is very important in increasing the bioavailability of health beneficial lycopene [96].

As tetrapyrroles, carotenoids are not synthesized by humans and other animals, and moreover they are required for their metabolism. Table 2 illustrates the health benefits obtained with carotenoids from phytoplankton.

**Table 2:** Bioactivities of carotenoids from microalgae and cyanobacteria.

Pigments	Bioactivities	References
Aphanizophyll	Photoprotection	[97,98,99]
Astaxanthin	Anti-allergic, anti-cancer, anti cardiovascular diseases	[100,101,102]
	Anti-oxidant	[100,103,104]
	Photoprotection	[38,105]
Canthaxanthin	Anti-cancer	[106]
	Anti-oxidant	[107]
β-Carotene	Anti-allergic	[108,109]
	Anti-cancer	[110,111]
	Anti-oxidant	[112]
β-Cryptoxanthin	Anti-inflammatory	[113]
	Improvement of skin health	[114]
Diadinoxanthin/Diatoxanthin	Photoprotection	[115,116]
Fucoxanthin	Anti-allergic	[109,117,118]
	Anti-cancer	[119,120]
	Anti-inflammatory	[121,122,123]
	Anti-obesity	[124,125,126,127]
	Anti-oxidant	[128,129]
	Photoprotection	[130]
Fucoxanthinol	Anti-cancer	[131]
	Anti-obesity	[124,125,126]
Lutein	Anti-inflammatory	[113]
	Protection of eyes	[131,132,133]
Myxoxanthophyll	Anti-oxidant	[134,135]
	Photoprotection	[97,98,99]
Neoxanthin	Anti-cancer	[134]
Orange Carotenoid Protein	Photoprotection	[136,137,138,139]
Peridin	Anti-cancer	[140]
Sporopollenin	Photoprotection	[141]
Synechoxanthin	Anti-oxidant	[142]
Violaxanthin	Anti-cancer	[111,112,134]
Violeaxanthin	Anti-cancer	[111,112,134]
Zeaxanthin	Anti-allergic	[109]
	Antioxidant	[135,143]
	Photoprotection	[139]

##### 3.1.2.1. Anti-Oxidant, Anti-Inflammatory and Anti-Mutagenic Activities

Number of works report anti-oxidant properties of microalgae and cyanobacteria [107,110,143]. Most microalgal and cyanobacterial pigments exert strong anti-oxidant activities *in vitro* and *in vivo* in animal models (Table 2). It was postulated that circulating anti-oxidants also have a role in the prevention of cardiovascular disease: C-reactive protein and oxidized low-density lipoprotein-cholesterol concentrations, are inversely related to plasmatic concentrations of vitamin C, carotenoids and phenols [144,145,146].

Therefore, dietary anti-oxidants may be protective against the development of inflammatory disease. However, additional intervention trials are required with carotenoids to precise their absorption, metabolism and potential as natural anti-oxidant, anti-inflammatory and anti-mutagenic compounds *in vivo*. Carotenoids have also a role in regulating gene expression and in inducing cell-to-cell communications [147].

Astaxanthin (Asta) is a nonprovitamin A found in *Haematococcus pluvialis*, up to 2–20 g kg^−1^ on a dry weight basis [148]. It is a powerful anti-oxidant, more effective than vitamins C and E or other carotenoids [149,150]. In particular, it has been reported that the anti-oxidant properties of Asta are about 10 times greater than those of β-carotene, lutein, zeaxanthin, canthaxanthin and over 500 greater than that of α-tocopherol. Thus the term “super vitamin E” has been proposed for Asta [100,103]. Asta has also a great potential to prevent cancer, diabetes and cardiovascular diseases [100,101]. The presence of the hydroxyl and keto endings on each ionone ring explains Asta unique features, such as the ability to be esterified [115], a higher anti-oxidant activity and a more polar configuration than other carotenoids [38,102,128]. Because of its anti-oxidant and membrane preservation properties, Asta has a considerable potential in the prevention and treatment of various chronic inflammatory disorders, such as cancers, rheumatoid arthritis, metabolic syndrome, diabetes, diabetic nephropathy, and gastrointestinal liver and neurodegenerative diseases, and could provide benefits not only for the cardiovascular system, but also in other inflammatory diseases. Therefore, its daily consumption is a practical and beneficial strategy in human health management [101]. Despite of these studies the molecular mechanism related to the biological activities of Asta is not yet fully understood. In a recent study, Sayo *et al.* [114] investigated whether the actions of nonprovitamin A carotenoids including Asta are mediated *via* retinoid signaling in cultured human keratinocytes. They found that β-carotene, β-cryptoxanthin, lutein, zeaxanthin, and Asta, upregulated *HAS3* gene expression and stimulated hyaluronan synthesis. The results suggest that nonprovitamin A can be substituted for retinoids and should be considered as a potential means of improving skin health. The several examples of benefits brought by Asta on health have however to be verified using human trials. To our knowledge, such trials have been started. For instance, Djordjevic *et al.* [104] tested the effect of Asta supplementation on muscle enzymes, oxidative stress markers and antioxidant response in elite young soccer players in Serbia. The results suggest that soccer is associated with excessive production of ROS and oxidative stress, which are prevented in Asta-supplemented in young soccer players.

Fucoxanthin (Fuco) is a brown pigment belonging to the class of xanthophylls, with anti-oxidant properties [128] under anoxic conditions, whereas other carotenoids have practically no quenching abilities, donating electrons as a part of its free-radical quenching function [129]. Fuco protects cells and provides other health benefits [38]: it improves cardiovascular health, blood pressure, and liver functions; it reduces inflammation, cholesterol and triglyceride levels [119,121,124]. Moreover, Fuco has anti-inflammatory effects via inhibitory effects of nitric oxide production [122,123].

Future clinical studies should determine the effectiveness of Asta and Fuco, not only on the vascular structure, but also on cartilage and joint health in at-risk patients or in those with established osteoarthritis [38].

β-Carotene isan important carotenoid because it displays a pro-vitamin A activity. Because animal cells cannot synthesize this molecule, it is considered as an essential compound in the human body [151].

Canthaxanthin has also anti-cancer properties [106] and a potent antioxidant. In particular, it inhibits the oxidation of lipids in liposomes [107].

Lutein and β-cryptoxanthin may down-regulate factors involved in inflammation associated with osteoarthritis and rheumatoid arthritis [113]. Lutein is currently considered as effective agent for the prevention of a variety of human diseases. Synechoxanthin, isolated in 2008 from the cyanobacterium *Synechococcus* sp. strain PCC 7002, has exceptional strong antioxidant properties [142].

##### 3.1.2.2. Anti-Cancer

Epidemiologic studies demonstrate an inverse relationship between cancer incidence and dietary carotene intake or blood carotenoid levels, provided the carotene intake is not too high [140,152]. For instance, Moreau *et al.* [120] works focalized to carotenoids extracts of the marine diatom *Odontella aurita* that showed an anti-proliferative effect on cultures of bronchopulmonary and epithelial cells when extracts were added to cell medium. Carotenoids such as astaxanthin or lycopene are active inducers of communication between cells at the cell-gap junctions [100,102,153]. DNA has receptors for specific carotenoids in controlling connexin gene transcription (connexin being a protein that mediates inter-cellular permeability and communication). The immune system cells need intercellular communication to function effectively, so they can be affected by the DNA regulating mechanism of carotenoids. For example, high doses of β-carotene increase the CD4 to CD8 lymphocyte ratio [154]. MDR reversion is often observed. Microalgae pigments may have interest to restore drug sensitivity or reverse MDR in cancer cells, as some of them inhibit or down-regulate drug efflux pumps. As examples, neoxanthin increases rhodamine accumulation in MDR colon cancer cells [134], inhibits the P346 glycoprotein efflux pump and reverses MDR in doxorubicin-resistant MCF-7 cells and hmdr1-transfected L1210, at 4 and 40 μg mL^−1^, respectively [155]. Violaxanthin and violeoxanthin are effective MDR modulators in colon, at 4 and 40 μg mL^−1^, respectively [111,112,134].

##### 3.1.2.3. Anti-Allergic

Some experiments performed on mices demonstrated that carotenoids (α- and β-carotene) can reduce sharply the immunoglobulin E production. For instance, the reduction of food allergy was observed on animals sensitized to ovalbumine [156]. Another work reported a similar effect of β-carotene combined with vitamin E on splenocytes [108]. Fuco, especially, but also Asta, zeaxanthin and β-carotene have anti-allergic propreties; they can block the aggregation of high affinity receptor for immunoglobin E via inhibiting their translocation to lipid rafts [109]. Fuco anti-allergic activity was recently evidenced using a rodent mast cells model [117,118].

##### 3.1.2.4. Anti-Obesity

Fuco and its metabolite fucoxanthinol [135] limit the risk of obesity [124,125]. Actually, these two marine carotenoids inhibit lipase activity in the gastrointestinal lumen and suppress triglyceride absorption [126]. During normal metabolism, the body produces heat [38]: fuco affects many enzymes involved in fat metabolism determining an increase in the release of energy from fat [127], thus an increase of thermogenesis.

Treatment with neoxanthin significantly reduces lipid accumulation. Examination of structures and functions suggests that carotenoids containing an allene bond and an additional hydroxyl sustituent on the side group may show suppressive effects on adipocyte differentiation [157].

##### 3.1.2.5. Protection against Light

To cope with the deleterious effects of excess illumination, photosynthetic organisms have developed photoprotective mechanisms that dissipate the absorbed excess energy as heat from the antenna system. Certain bloom-forming microalgae are “sun-adapted” and have the capacity to synthesize sunscreen pigments [158]. In many microalgae, the cell is made more resistant to UV light by the accumulation in the cell wall of sporopollenin [141], a carotene-polymer absorbing UV light. Lutein is one of the major xanthophylls found in green microalgae. It selectively accumulates in the macula of the human retina, protects the eyes from oxidative stress, and acts as a filter of the blue light involved in macular degeneration and age-related cataract [131,132,133]. Asta protects against UV light; it is true for microalgae, animals and human beings [38,105]. Fuco inhibits tyrosinase activity, melanogenesis in melanoma and UVB-induced skin pigmentation by topical or oral application [130].

In cyanobacteria, strong blue-green (or white) light activates a soluble carotenoid protein, the OCP which, by interacting with the phycobilisome, increases energy dissipation in the form of heat, thereby decreasing the amount of energy arriving at the RCs. Consequently, OCP plays a key role in the photoprotective mechanism in cyanobacteria [136,137,138,139] and it is the first photoactive protein identified containing a carotenoid as the photoresponsive chromophore [139]. The fluorescence decrease associated with this energy dissipation in the antenna was called qEcya to differentiate this mechanism from that existing in plants. The qEcya mechanism is not the only one that protects cyanobacteria cells from high irradiance. Mutants lacking zeaxanthin are very sensitive to strong light, even more than mutants lacking the qEcya mechanism, indicating an important role of this carotenoid in photoprotection [139].

Another protein, called the Fluorescence Recovery Protein (FRP) encoded by the *slr1964* gene in *Synechocystis* PCC 6803, plays a key role in dislodging the red OCP from the phycobilisome and in the conversion of the free red OCP to the orange, inactive, form. As a result, under low light conditions, the FRP mediates the recovery of the full antenna capacity by accelerating the deactivation of the OCP [159]. The amplitude of photoprotection depends on both OCP concentration and FRP concentration but also on the OCP/FRP ratio. Therefore, the synthesis of FRP and OCP is probably strictly regulated to accomodate both concentration and OCP/FRP ratio to the right level of photoprotection.

### 3.2. Lipids

Marine and freshwater microalgae are known to be characterized by a large diversity in their lipid and fatty acid composition. Glycerides, phospholipids (PLs) and glycolipids are major classes produced and stored in different subcellular compartments of microalgae such as globules, endoplasmic reticulum and chloroplast, respectively. The lipid composition in marine microalgae reveals that the major lipid classes are neutral lipids and glycolipids but also, even in a low range, the phospholipid class. According to this wide variety in lipid class, these microorganisms are good candidates to produce these lipids for which the amounts can vary according to culture and environmental factors.

Triacylglycerols (TAG) and diacylglycerols (DAG), monogalactosyldiacylglycerols (MGDG) and digalactosyldiacylglycerols (DGDG), and phosphatidylcholine (PC) and phosphatidylethanolamine (PE) are the main classes as constitutive of neutral lipids, glycolipids, and phospholipids, respectively. In this section will be described a non-exhaustive information concerning the use and the effects of these lipids in human or animal models in relation with their health benefits.

#### 3.2.1. Omega-3 Polyunsaturated Fatty Acids

Omega-3 Polyunsaturated Fatty Acids (PUFA) are well known to be beneficial for human health. The most recognized omega-3 fatty acid source is fish oil but its quality depends on several parameters such as species, seasons and quality of consumed food. Moreover, the content of lipid-soluble pollutants in fish oil may be high because of the biomagnification of such compounds in food chains. Microalgae, and specifically the marine strains, are considered as more interesting alternative sources for omega-3 PUFA production. Indeed, they have PUFA of higher and more constant quality than the ones from fish because they are at the bottom of food chains.

Among the marine strains, the prymnesophytes as *Isochrysis galbana* and *Pavlova lutheri* are rich sources of docosahexaenoic acid (DHA; 6%–14% total fatty acids) and eicosapentaenoic acid (EPA; 5%–26% of the total fatty acids [160,161,162,163,164], while diatoms as *Phaeodactylum tricornutum* or *Odontella aurita* are rich sources of EPA (26% of the total fatty acids) [160,161,165].

Due to its content of DHA and EPA, *Isochrysis galbana* was used in alloxan-induced diabetic rats. This microalga induced a decreased glucose, TAG and cholesterol blood levels. However, an increased light-density lipoprotein level and a decreased high-density lipoprotein level were observed in the diabetic rats, but also in the healthy ones [166]. The marine diatom *Odontella aurita* is the only microalga known to be rich in EPA and currently approved as a dietary supplement [120].

Omega-3 fatty acids act as important mediators of gene expression working via the peroxisome proliferator-activated receptors that control the expression of the genes involved in the lipid and glucose metabolism and adipogenesis [167]. Ethyl-EPA markedly reduced the fatty droplets in the liver cells of mice fed a high-fat diet, also lowering plasma levels of total cholesterol and TAG [168]. Kajikawa *et al.* [169] showed that oral administration of highly purified EPA ethyl ester improved hepatic fat accumulation in high fat/high sucrose diet-fed mice by suppressing the TAG synthesis enzymes regulated by sterol regulatory element binding protein-1 and decreased the accumulation of hepatic monounsaturated fatty acids produced by stearoyl-coenzyme A desaturase 1. Numerous studies showed biochemical functions of *Chlorella*, especially when administered on the presence of underlying disorders such as in streptozotocin induced diabetes rats [170]. Recent studies in hypercholesterolemic rats have shown that DHA supplementation reduced total weight gain, adiposity index, high-density lipoprotein (HDL)-cholesterol and glucose plasmatic concentration regardless of the dose and form of supplementation [171].

##### 3.2.1.1. Cardioprotective Effects

The cardioprotective effects of the omega-3 fatty acids such as EPA and DHA has been reported through epidemiological studies in human and animal models but also in cell culture studies [172,173,174,175]. Fish oil supplements, and other sources such as microalgae provide EPA and DHA usually used for human diets [176,177]. Omega-3 fatty acids are known to reduce the risk of cardiovascular disease, through a reduction of factors involved in metabolic syndrome development, by modification of serum lipid profile [176].

##### 3.2.1.2. Anti-Obesity

EPA and DHA are involved in a reduction of TAG synthesis and adiposity. During obesity, the body mass index and waist circumference are inversely correlated with EPA and DHA intakes [178]. In rabbits fed with a high cholesterol diet for 10 weeks, *Chlorella vulgaris*, one species of *Chlorella*, showed anti-lipidemic and anti-atherosclerotic actions [176]. Cherng *et al.* [179] also showed that *Chlorella pyrenoidosa* has the ability to prevent dyslipidemia in rat and hamster models that were fed chronic high fat.

##### 3.2.1.3. Anti-Cholesterol

In type 2 diabetes patients, dietary EPA and DHA have been shown to reduce TAG and very low-density lipoprotein-cholesterol levels and to increase the HDL-cholesterol level, in blood [180]. Patients with dyslipidemia have been shown to have significant decrease in blood TAG and increase in HDL-cholesterol after a consumption of EPA and DHA during 3 and 6 months [181]. Adan *et al.* [182] showed that EPA and DHA feeding reduces serum cholesterol and TAG levels, and decreases platelet aggregation in hypercholesterolemic rats. Ethyl esters of DHA extracted from microalgae have been reported to decrease the plasma TAG and total cholesterol levels in rats fed high-fructose diet [183]. Another observation has indicated that *Chlorella* intake can reduce cholesterol levels in patients with hypercholesterolemia [184].

##### 3.2.1.4. Normalizing Platelet Hyper-Aggregability

EPA and DHA also play a key role in normalizing platelet hyper-aggregability. When added to the diet, EPA and DHA can alter the phospholipid membrane composition of the cells, and therefore impact on the synthesis and action of eicosanoids, and regulate transcription factor activity and abundance [177]. During dyslipidemia in rats, the use of the marine diatom *Odontella aurita* has shown beneficial effects as a reduction in the risk factors for high-fat induced metabolic syndrome such as hyperlipidemia, platelet aggregation and oxidative stress [177].

#### 3.2.2. Glycerides

TAG are beneficial to health provided they are rich in PUFA. This is the case for instance in the marine dinoflagellate *Crypthecodinium cohnii* that can accumulate a high % of DHA (25%–60% of the total fatty acids) in its TAG (oil) with only trivial amounts of other PUFA [185,186]. DAG may be also interessant although considered as minor constituent [187]. Several studies have been conducted using a DAG-oil (producted during high-temperature process), in which the 1,3-DAG isoform was the major component. In this case, and by comparison with a TAG rich oil, a suppression of hypertriglyceridemia during the post-prandial period was observed. Moreover, a decreased body fat mass was obtained [188,189,190]. In rodent models, the anti-obesity effect of DAG oil was attributed to an increased fatty acid beta-oxidation and to a suppression of TAG synthesis [191,192]. 

#### 3.2.3. Phospholipids

Phospholipids are the most important storage forms of the biologically active PUFA. In marine sources such as fish or microalgae, the omega-3 fatty acids are usually found both in triacylgycerol form and as phospholipids. Many studies have reported high amounts of omega-3 fatty acids such as EPA and DHA in phospholipids from fish and other marine sources [193,194,195,196]. Omega-3 fatty acid rich PC by comparison with other PC improves disorders related with obesity. Indeed, these phospholipids increase the fatty acid beta-oxidation and the adiponectin levels in serum of obese rats [197].

In addition, Lemaitre-Delaunay *et al.* [198] have shown the potential interest of PC-DHA over TAG-DHA, which might favor the DHA uptake by erythrocytes and putatively by the brain, providing that phospholipid sources of DHA are available. Indeed, the metabolic fate of DHA differs substantially when ingested in TAG or PC both in terms of bioavailability in plasma and accumulation in target tissues.

The intake of dietary phospholipids such as PC and PE are estimated to 3–4 g/day, which amounts 5%–8% of total dietary lipids [199,200]. These amounts are enough to have beneficial effects on health, by comparison with dietary TAG. PE intake has been reported to have a cholesterol-lowering effect in rats in relation with fecal neutral steroid excretion [201,202]. Cholesterol liver metabolism is also improved by PC. Indeed, this phospholipid has protective effects on liver metabolism; it has been observed a decreased cholesterol level by enhancing bile cholesterol secretion, decreasing lymphatic cholesterol absorption and reducing fatty acid synthesis in different animal models [203,204]. Minor phospholipids such as phosphatidylinositol has been reported to increase the HDL-cholesterol levels in mammals [205,206,207], by mobilization of cellular sterol.

## 4. Changing the Cultivation Conditions for Optimizing the Production of Bioactive Compounds

Abiotic stresses, such as high irradiance, nutrient starvation, UV irradiation, trigger metabolic reorientations ending with the production of other bioactive compounds such as omega-3 fatty acids or carotenoids.

### 4.1. Pigments

In natural conditions, the pigment contents vary with hydrographic factors of the ocean and the seasons (e.g., [208]). It means that cultivated algae can produce large quantities of pigments if some environmental growth conditions, such as temperature, light intensity and nutrients, are controlled [209].

#### 4.1.1. Tetrapyrroles

In the photosynthetic membranes, closed tetrapyrroles form complex with carotenoids. In these complexes, they are photoprotected from photooxidation by carotenoids [68,210]. This protection is absolutely required because the absolute yield of singlet oxygen formation through tetrapyrrole irradiation (via its triplet excited state) is between 0.3 and 0.9, depending on the type of tetrapyrrole and solvent conditions (reviewed by Spikes and Bommer [211]. Singlet oxygen formation triggers a cascade of reactions in which other ROS are formed [212], reviewed by Spikes and Bommer [211]). Photodynamic herbicides (reviewed by [213]) and photodynamic treatments of cancers (reviewed by [214,215]) are based on this mechanism. Therefore, tetrapyrrole synthesis and accumulation in photosynthetic cell was always strictly regulated and even for short illumination time, tetrapyrroles should be protected [37,216,217]. Consequently, the tetrapyrrole biosynthetic pathway will not be reviewed in this contribution.

#### 4.1.2. Carotenoids

**Figure 1:**
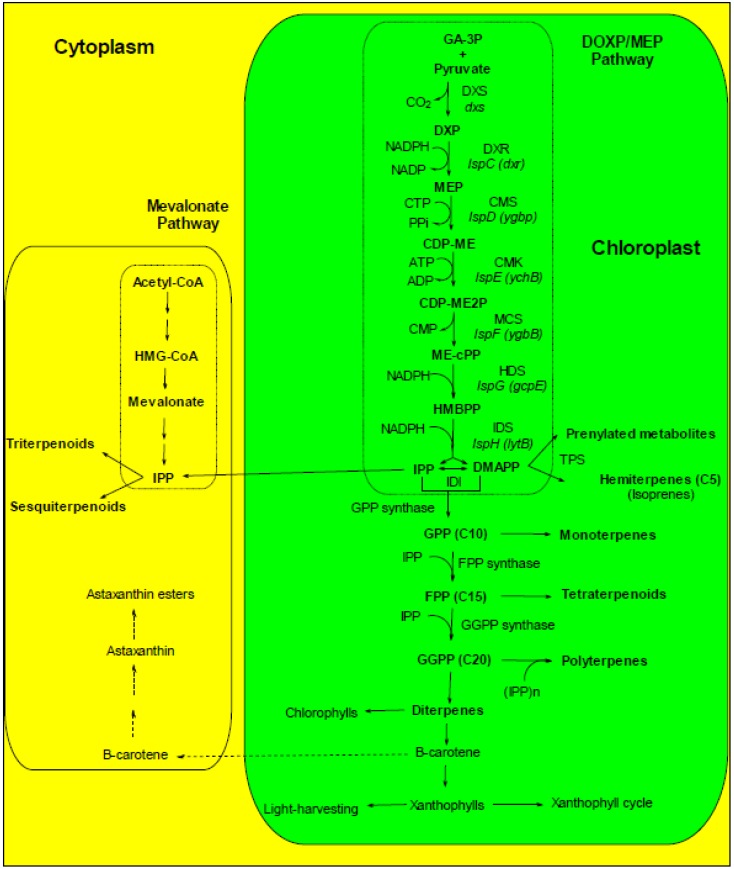
Scheme of the biochemical pathways leading to carotenoids and other terpenoids. The DOXP/MEP pathway produces the isoprenoid precursors DMAPP and IPP, that are required for the production of carotenoids and other chloroplast-produced terpenoids. Some of the intermediates of the DOXP/MEP pathway can be exported to the cytoplasm and used in the mevalonate pathway of terpenoid synthesis. The cytoplasmic mevalonate pathway does not provide IPP to the chloroplastic DOPX/MEP pathway neither in chloroplast nor in chromoplasts [231]. In contrast, the chloroplastic pathway delivers IPP to the cytoplasmic pathway, allowing the incorporation of photosynthetically fixed C into sterols [231]. Dashed arrows indicate the Asta biosynthetic pathway from β-carotene.

In microalgae, photosynthesis is efficiently performed if carotenoids are bound to peptides to form pigment–protein complexes in the thylakoid membrane. In algae, carotenoids function essentially as accessory light harvesting pigments during the light phase of photosynthesis and as agents to photoprotect the photosynthetic apparatus from excess light dissipation by scavenging ROS such as singlet oxygen and free radicals [218,219,220]. Carotenoids in the majority of green algae are synthesized and accumulate within plastids, while secondary xanthophylls (e.g., Asta in *Haematococcus pluvialis*) accumulate in the cytosol. However, these xanthophylls are synthesized in the chloroplast, but they accumulate in the cytosol (Figure 1). Thus xanthophylls can be found in all cellular compartments [221]). NADPH is required in the early steps of carotenoid biosynthesis [222]; consequently, for an efficient carotenoid biosynthesis, efficient light-dependent reactions of photosynthesis are needed (NADP^+^ must be photoreduced) (Figure 1).

Most of the carotenoids of cyanobacteria are embedded in the photosynthetic membrane which is both, the site of their synthesis and the site of their major functions of light harvesting and photoprotection. Some carotenoids are exported to the periphery of the cell, especially during high intensity light exposure, where it may provide additional photoprotection [223,224]. Other carotenoid proteins may be involved in the transport of carotenoid from the photosynthetic membrane to the exterior.

Control of photosynthetic gene expression and enzyme activity is coordinated by a number of molecular triggers including photosynthetic metabolites, hormones, Chl and carotenoid biosynthesis precursors, photoreceptors, stress-generated ROS, and soluble redox active compounds [225,226]. All known enzymes involved in carotenoid synthesis are nuclear encoded, but the carotenoid synthesis itself takes place in chloroplasts [222,227,228] (Figure 1). Most of xanthophylls are structural and functional components of the cellular photosynthetic apparatus and for this reason are considered as primary carotenoids essential for survival [93] whereas it is not the case for secondary carotenoids produced in large amounts [93,229,230]. Carotenoid content in some microalgae can be increased up to 140 mg g^−^^1^ biomass by manipulating environmental conditions [149].

CDP-ME: 4-diphosphocytidyl-2-C-methylerythritol; CDP-ME2P: 4-diphosphocytidyl-2-*C*-methyl-d-erythritol-2-phosphate; CMK: CDP-ME kinase; CMS: CDP-ME synthase; DMAPP: dimethylallyl diphosphate; DOXP: 1-deoxy-d-xylulose 5-phosphate; DXR: DOXP reductoisomerase; DXS: DOXP synthase; FPP: farnesyl diphosphate; GA-3P: glyceraldehyde-3-phosphate; GGPP: geranylgeranyl diphosphate/pyrophosphate; GPP: geranyl diphosphate; GPPS: geranyl diphosphate synthase; HDS: HMBPP synthase; HMBPP: (*E*)-4-Hydroxy-3-methyl-but-2-enyl pyrophosphate; IDI: isopentenyl/dimethylallyl diphosphate isomerase; IDS: IPP/DMAPP synthase; IPP: isopentenyl diphosphate; MCS : ME-cPP synthase; ME-cPP: 2-*C*-methyl-d-erythritol 2,4-cyclopyrophosphate; MEP: 2-*C*-methyl-d-erythritol-4-phosphate.

##### 4.1.2.1. Temperature

Increasing the temperature is a mean to change the polar to non-polar carotenoid ratio. Examples can be found more especially with cyanobacteria. For instance Klodawska *et al.* [232] demonstrated it on the cyanobacterium *Synochocystis* sp. PCC6803, for which polar to non-polar xanthophyll ratio increased from 25 °C to 35 °C. Another example is given by Mehnert *et al.* [97] who found a specific increase of echinenone content in the cyanobacteria *Cylindrospermopsis raciborskii* and *Aphanizomenon gracile* when cultivated from 10 °C to 35 °C. In both examples, the membranes of the living cells became more rigid with the increasing of temperature.

##### 4.1.2.2. Light Intensity

Microalgae and cyanobacteria are confronted with variations of light by movements in the water column and emersion for coastal benthic species. For these reasons, more than terrestrial plants, marine microorganisms acclimate to light level and light quality by optimizing pigment content and composition [208,233,234,235]. Therefore they display a wide diversity of light harvesting pigments (Table 1) allowing optimal photosynthesis at different depths in seawater. 

Autotroph microorganisms sometimes experience high levels of UV-radiation, in shallow areas and during low tides when they are deposited on intertidal flats. To cope with high sunlight intensities, microalgae have developed different photoprotective mechanisms. One of these, the xanthophyll cycles (Figure 1), consists in the reversible conversion of violaxanthin, antheraxanthin and zeaxanthin in green algae and in the reversible conversion diadinoxanthin and diatoxanthin in brown algae [115,234,236]. Several authors have shown that UV exposure increases carotenoid content in the following hours. It is true for microalgae [237,238,239] and cyanobacteria [98,240]. In cyanobacteria, carotenoid glycosides play an important role in photoprotection [97]). In cyanobacteria submitted to high light and nitrogen deprivation, the carotenoid content can increase [241]. A high light stress alone leads to an increase of myxoxanthophyll and aphanizophyll, and to a decrease of β-carotene content [97,98,99]. The increase of myxoxanthophyll amount is in agreement with a coordinated activity of the enzymes of the myxoxanthophyll biosynthetic pathway, with no rate-limiting intermediate steps [99]. 

Acclimation of microalgae or cyanobacteria to low irradiance intensity or blue enriched light increases the synthesis of specific carotenoids (e.g., fuco in phaeophyceae [196,208,241]). Generally, the photoprotection or the low photoacclimation leads carbon to carotenoid synthesis whereas in nonstressfull conditions, carbon serves mainly to growth (cell wall edification). In the marine diatom *Haslea ostrearia*, carbon fixation by β-carboxylation is almost equal to that in the C3 pathway whereas under low irradiation C3 fixation dominates [242].

##### 4.1.2.3. Nutrients

Nutrient starvation is generally efficient for the overproduction of carotenoids in cyanobacteria [243]. It is also the case for microalgae. For instance, nitrogen starvation triggers β-carotene in *Eustigmatos* cf. *polyphem* (Eustigmatophyceae) [244] and in *Dunaliella salina* [245]. β-carotene can accumulate in lipid globules until 14 mg per liter cell volume (*i.e.*, 2.7% of ash-free dry weight). Actually, Lamers *et al.* [245] applied a combined high light and nitrogen stress and observed a β-carotene accumulation which negatively correlated with the degree of insaturation of the total fatty acid pool. In a frame of an optimal production of biofuels by microalgae, nitrogen starvation is often applied and leads at the same time to an overproduction of β-carotene. Nitrogen starvation leads to a modification of the photosynthetic apparatus. The linear electron flow from water to NADP^+^ was slowed down while the cyclic electron flow was increased [246]. An overproduction of β-carotene can also be obtained with high nitrate contents (1–3 mM) in *Dunaliella* *salina* [247,248].

Low nitrate level, coupled with high light intensity, was the key to high cellular accumulation of Asta in *Haematococcus* sp [249,250] or *Chlorella* sp [251]. Chloroplasts of *H. pluvialis* degenerate in the transition from green coccoid cells to red cyst cells during encystment. Oil droplets containing Asta accounted for more than 50% of the cyst cell volume [252].

Varying the salt concentration is an alternative to get more carotenoids. For instance, when grown under optimized conditions of salinity and light intensity, *Dunaliella* sp produces up to 14% β-carotene [149,253,254]. The culture of the cyanobacterium *Synechocystis* sp. PCC 6803 in high-salt conditions (0.7 M NaCl) triggers the up-regulation of protective carotenoid pigments, especially zeaxanthin and myxoxanthophyll [255]. The presence of externally added trehalose at the same time of the salt stress increased even harder the carotenoid to Chl *a* ratio in this strain. The high accumulation of carotenoids in the presence of trehalose could allow a best cell protection against salt induced production of ROS, and thus a best cell protection against oxidative damage [255].

##### 4.1.2.4. Other Stress

As carotenoids are thought to play an important role in the protection of cellular membrane lipoproteins against oxidative damage by scavenging several active oxygen species, heavy metal stress may stimulate carotenogenesis in cyanobacteria. Actually, elevated levels of β-carotene and Asta were observed in the cyanobacteria *Hapalosiphon fontinalis* in response to chromium (VI) [256].

Cells of *Anabaena* sp. PCC 7120, a low desiccation tolerant cyanobacterium, was subjected to prolonged desiccation and effect of loss of water was examined on antioxidant response as well as on overall viability in terms of photosynthetic activity. In desiccated state, Chl *a* and carotenoid contents increased after 1 h of incubation under desiccation. The oxygen-evolving activity declined in desiccated cyanobacterial biomass while rehydration led to instant recovery, indicating that cells protect the photosynthetic machinery against desiccation [257]. As desiccation leads to oxidative stress, an enhanced level of carotenoids most likely facilitates quenching of ROS.

### 4.2. Lipids

#### 4.2.1. Temperature

The effects of temperature on growth and on lipid and fatty acid metabolism have been studied on several microalgae. Indeed, in natural conditions, the temperature is one of the main environmental factors that can influence the biology of microalgae, and thus, influencing the carbon metabolism.

In microalga *Pavlova lutheri*, it has been shown that the decrease of growth temperature (from 25 to 15 °C) has the consequence to increase the polar lipids such the betaine lipids [163]. In these conditions, the levels of sulfoquinovosyldiacylglycerol and phosphatidylglycerol were increased while the MGDG and TAG levels were decreased. Concerning the omega-3 fatty acids, the EPA and DHA levels were also increased. These results are considered as an adaptative response to temperature with a modulation of omega-3 fatty acids incorporated into lipids involved in the membrane building.

In the diatom *Phaeodactylum tricornutum*, the decrease of growth temperature (from 25 to 10 °C) showed that the representative fatty acids in the total fatty acids decreased proportionally by about 30% in palmitic acid and 20% in palmitoleic acid but increased about 85% in EPA [258]. Other species such as the Prymnesiophyte *Isochrysis galbana*, when cultured at temperatures from 15 to 30 °C showed a global lipid accumulation, with a slight decrease of the neutral lipids, by comparison with other polar lipids. The glycosylglyceride levels were increased while no change was observed concerning the phosholipid classes. At 15 °C, as observed for other strain, higher levels of omega-3 fatty acids were obtained (α-linolenate and DHA) while the monounsaturated and saturated fatty acid levels were decreased [259]. Concerning the omega-3 fatty acid levels (EPA and DHA), they were lower at the highest temperatures [260]. The diatom *Chaetoceros* sp., a tropical Australian microalga, was studied at different growth temperatures, from 25 to 35 °C. The highest lipid percentage was obtained when the cells were cultured at 25 °C. Authors showed that the highest omega-3 fatty acid levels were slightly lower at the highest temperatures [260,261]. The same observations were made concerning *Phaeodactylum tricornutum* in which the saturated, monounsaturated but also the omega-6 fatty acids were increased with temperature, while the EPA level was decreased [262]. Lipids produced at varing temperatures can have varing biological activities such as antibacterial and cytotoxic activitiesas reported by Gacheva *et al.* [263] for the fatty acids from *Gloeocapsa* sp.

#### 4.2.2. Light Intensity

Light intensity level is the major parameter involved in the microalgal metabolism, in relation with photosynthesis activity. Several studies have been conducted in *Pavlova lutheri*, a prymnesiophyte rich in both EPA and DHA. The proportions of PUFA, especially EPA, have been shown to be significantly higher under low light, and saturated fatty acids and DHA levels were significantly higher under high light. In this microalga, the galactolipids, a major component of chloroplast lipid membranes, made up approximately 54%–66% of total lipids. The highest PUFA levels, such as those of EPA, were predominantly found in the galactolipid fraction when the cells were grown at low light [264]. Using radiolabelling carbon sources as substrate, the response to light intensity was specific of the use of hydrogenocarbonate or acetate [265]. Indeed, it has been shown that the lipid synthesis, including galactolipids and phospholipids, was increased with light intensity when the microalgae used inorganic carbon (hydrogenocarbonate) but a less sensitivity was observed to the light intensity changes when acetate, as carbon source, was used. Concerning the omega-3 fatty acids, EPA and DHA synthesis has been shown to be lower with high light intensities with hydrogenocarbonate but not with acetate. These results led the authors to hypothesize the fact that *Pavlova lutheri* was able to synthesize LC-PUFA by two distinct pathways: one inside the chloroplast, depending on light intensity, and another one outside the chloroplast, independent of light.

Studies of light intensity were made with the diatom *Thalassiosira pseudonana* showing significant changes in fatty acid and lipid composition. Cells grown under moderate continuous light level showed high accumulation of TAG and a reduced level of polar lipids. In the diatom *Skeletonema costatum*, the highest levels of EPA are observed with the highest light levels during late exponential phase and during the stationary phase [266]. In *Nitzschia closterium* and *Isochrysis zhangjiangensis*, after a 3 day-UVA-stress period, the PUFA content increased too [239]. By opposition, in *Nannochloropsis* sp., the degree of unsaturation of fatty acids decreased with increasing irradiance, with a 3-fold decrease of the percentage of total omega-3 fatty acids, caused mainly by a decrease of EPA [267]. Dark exposure can also impact the lipid content; for instance, the dinoflagellate *Prorocentrum minimum* reduces its content of TAG and galactolipids, whereas the total content of phospholipids changed little (PC, PE and phosphatidylglycerol decreased; phosphatidylserine, phosphatidic acid and phosphatidylinositol increased) [268].

#### 4.2.3. Nutrients

Other factors such as nutrients are involved in lipid and fatty acid synthesis. A well-documented description has been reviewed by Guschina [269]. The effect of phosphorus-limitation has been studied in different species of marine algae [270]. It has been reported that phosphorus-limitation led to an increased lipid content in several species such *Phaeodactylum tricornutum*, *Chaetoceros* sp. and *Pavlova lutheri*, while a decreased lipid content in the green flagellates, *Nannochloris atomus* and *Tetraselmis* sp. was observed. The effect of nitrogen-limitation has been studied in the microalga *Phaeodactylum tricornutum* [271]. A decrease of the nitrogen concentration caused the decrease of the galactolipid fraction from 21% to 12%. In contrast, both neutral lipids and phospolipids increased from about 73% to 79% and from 6% to 8%, respectively in nitrogen starved cells.

The effect of CO_2_ on the content and composition of lipid fatty acids and chloroplast lipids has been studied in *Dunaliella salina* [272]. An increase in the CO_2_ concentration from 2% to 10% was shown to increase the total fatty acid levels. Differences in the fatty acid content and composition indicated increased fatty acid synthesis *de novo* but an inhibition of their elongation and desaturation steps. This led to an increase in the relative content of saturated fatty acids at the highest levels of CO_2_. After CO_2_ stress, the MGDG/DGDG ratio was increased while the ratio of omega-3/omega-6 fatty acids, was clearly increased in the phosphatidylglycerol class.

*Dunaliella salina* is also tolerant to high salt. It has been shown that the expression of β-ketoacyl-coenzyme A synthase (which catalyzes the first and rate-limiting step in fatty acid elongation) increased in *Dunaliella salina* cells transferred from 0.5 to 3.5 M NaCl [273]. It has been suggested that the salt adaptation in this microalga induced modifications of the fatty acid composition of algal membranes, and lipid analysis indicated that microsomes, but not plasma membranes or thylakoids, from cells grown in 3.5 M NaCl contained a considerably higher ratio of 18C (mostly unsaturated) to 16C (mostly saturated) fatty acids compared with cells grown in 0.5 M NaCl. The salt-induced ketoacyl-CoA synthase, jointly with fatty aciddesaturases, was thought to play a role in adapting intracellular membrane compartments to function in the high internal glycerol concentrations used to balance the external osmotic pressure created by high salt [273].

## 5. Role of Chloroplast and Reticulum Compartments in Lipid and Fatty Acid Synthesis in Marine Microalgae

Marine microalgae can be considered as good models in lipid and fatty acid synthesis studies. Indeed, different works have described that in microalgae, the lipid and fatty acid metabolic pathways were distributed according to the location of enzymatic activities involved in their synthesis. The main compartments involved in lipid and fatty acid synthesis are chloroplast and endoplasmic reticulum in which the glycerolipid and phospholipid classes are recovered respectively. Concerning the phospholipid classes, their presence depends on the microalgal species. Indeed, phospholipids can be replaced by betaine lipids and thus gives some specificity in the concerned species. However, as for the phopholipids, the betaine lipids are also recovered outside the chloroplast, in the reticulum.

In this section, we will describe the location of the different metabolic pathways by the use or data reported on microalgae. Moreover, and specifically in the marine microalgae, the influence of environmental factors on omega-3 fatty acid metabolism will be described.

### 5.1. Lipid and Fatty Acid Plastid Synthesis in Microalgae

Main reports have been conducted in plant models from which lipid trafficking pathways have been proposed with a difference in lipid location. Even if the lipid composition of plant cell membranes can vary according to environmental factors, cell type culture or development state, it is recognized that glycolipids such as MGDG, DGDG or sulfoquinovosyldiacylglycerol are exclusively lipid elements of plastidial membranes while the phospholipids have a less typical distribution. Among the phosholipid classes, the PC, absent from the thylakoids, are only present in the outer membrane of plasts. Concerning the PE and phosphatidylserine, these classes are totally absent of the plasts [120]. From these results, it can be suggested that the enzyme complex involved in the lipid synthesis are located in specific cellular compartments or that lipid transport can occur in cell to transfer lipids from one compartment to another one.

It is well-recognized that the major lipid class in the chloroplast compartment is the glycolipid class even if phosphatidylglycerol is the main phospholipid in this subcellular fraction. Fatty acids incorporated into glycolipids are related with their metabolism. Indeed, according to the chain length, fatty acids are synthesized during a *de novo* plastid metabolism or are imported from the endoplasmic reticulum before being incorporated into the glycolipids [274]. According to the identification of the enzymes involved in the glycolipid synthesis in the chloroplast, and their resemblance with the bacterial enzymes, the plast metabolic pathways were named prokaryotic pathway while the reactions involved in the endoplasmic reticulum were named the eukaryotic pathway. The prokaryotic pathway is characterized by properties to incoporate C16 fatty acids at the *sn-1* position. Some fatty acids synthesized in the plast compartment are exported to the endoplasmic reticulum and these are involved in the synthesis of lipids of non chloroplastic membranes. These fatty acids, and specifically the C18 ones, are more incorporated into the *sn-2* position, and are used to synthesize longer and more unsaturated fatty acids through the eukaryotic pathway [274].

Another example of fatty acid and lipid traffic between the endoplasmic reticulum and the chloroplast is that in some algal species, fatty acids incorporated into diacylglycerols of the endoplasmic reticulum compartment are metabolized into the chloroplast one to produce in situ diacylglycerols for the formation of chloroplast galactolipids. Then, once these fatty acids are incorporated into structural lipids of chloroplasts, they can undergo desaturation without the need for deacylation-reacylation [275]. These results confirmed previous studies in which it was shown that MGDG-bound plamitate was desaturated into palmitoleate in the cyanobacteria *Anabaena variabilis*, without any detectable deacylation and reacylation [276]. Experiments conducted in *Dunaliella salina*, using radioisotope labeling experiments, showed that MGDG and DGDG were more associated with desaturation of galactolipid-bound fatty acids, in relation with desaturase enzymes located in the chloroplast envelope [277].

In the marine diatom *Phaeodactylum tricornutum*, it has been reported the characterization of two cDNAs coding for a plastidial and microsomal delta-12 desaturase. According to these results a compartmentalization in the synthesis of the different fatty acids have been proposed with an specific incorporation of EPA into the galactolipid fraction of the plastidic compartment [277].

In *Pavlova lutheri* species, two unusual compounds, diacylglyceryl carboxyhydroxymethylcholine (DGCC) and diacylglyceryl glucuronide (DGGA), are identified in the lipid fraction. DGCC is enriched in palmitate and EPA, and DGGA contained predominantly oleate, docosapentaenoic and docosahexaenoic (DHA) acids. Analysis of subcellular membrane fractions demonstrated an accumulation of these lipids together with a betaine lipid, diacylglyceryl hydroxymethyl-*N*,*N*,*N*-trimethyl-β-alanine, in non-plastid membranes [278]. Using radiolabelled acetate as precursor of lipid and fatty acid synthesis, the authors suggested that the betaine lipid DGCC acted as a first acceptor of *de novo*-formed or exogenous fatty acids which undergo processing and redistribution. It was suggested that C18 and C20 fatty acids were individually transferred from cytoplasm to chloroplast, allowing the synthesis of eukaryotic MGDG without import of DAG.

In this microalga, it has been shown that it was able to synthesize long chain-polyunsaturated fatty acids (LC-PUFA) from radiolabelled carbon sources such as hydrogenocarbonate or acetate, by successive elongation and desaturation steps. Synthesis of lipids, including galactolipids and phospholipids/betaine lipids has also been demonstrated. According to light-dependent studies, two distinct enzyme pools involved in the LC-PUFA synthesis have been proposed, one light-dependent inside the chloroplast, and another one, light-independent and outside the chloroplast [279]. Using both acetate and hydrogenocarbonate, this microalga was able to synthesize LC-PUFA by successive desaturation and elongation steps. The use of both substrates induces different synthesized fatty acids. Indeed, from hydrogenocarbonate as substrate, the main synthesized fatty acids are C16 fatty acids and EPA, while with acetate, the main fatty acid was a C18. One hypothesis proposed by the authors was that the synthesis of acetyl-CoA from hydrogenocarbonate is mainly intra-chloroplastidic by opposition with the synthesis of acetate outside the chloroplast. Thus, fatty acids synthesized from chloroplastidic acetyl-CoA can be metabolized by enzymes of thylakoid such as desaturases. Using radiolabelled acetate as substrate, radioactivity incorporated into fatty acids is supposed to derived from cytosolic acetyl-CoA formed, hypothesizing a *de novo* fatty acids synthesis outside the chloroplast [279].

**Figure 2:**
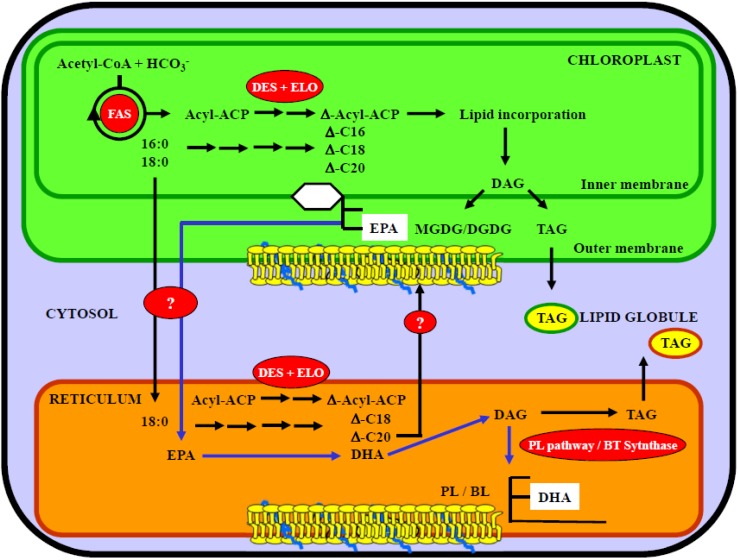
Proposed lipid synthesis pathway in the marine microalgae. Role of chloroplast and endoplasmic reticulum compartments in lipid synthesis and location of omega-3 fatty acid incorporation [265,277,278,279,280]. ACP, acyl carrier protein; BL, betaine lipid; DES, desaturase; DAG, diacylglycerol; DAGAT, diacylglycerol acyltransferase; DGDG, digalactosyldiacylglycerol; DHA, docosahexaenoic acid; ELO, elongase; EPA, eicosapentaenoic acid; FAS, fatty acid synthase; MGDG, monogalactosyldiacylglycerol; PL, phospholipid; TAG, triacylglycerol; Δ-Cx, unsaturated fatty acid.

A proposed lipid synthesis pathway in the marine microalga *Pavlova lutheri* is reported in Figure 2. The role of chloroplast and endoplasmic reticulum compartments is proposed for the lipid synthesis and the location of omega-3 fatty acid incorporation.

### 5.2. Compartmentalization of EPA and DHA Synthesis

Concerning the galactolipid synthesis and the incorporation of EPA, both prokaryotic and eukaryotic types have been shown to be the main end products of biosynthesis [281]. The prokaryotic molecular species, C20 fatty acids such as EPA and ARA were mainly incorporated into the *sn*-1 position of the galactolipids, while C16 fatty acids were preferentially esterified into the *sn*-2 one. Concerning the eukaryotic species, both positions were occupied by the C20 fatty acids [281]. Independently, whatever the molecular species, the cytoplasmic and chloroplastic lipids were involved in the metabolism of galactolipids. Cytoplasmic linoleoyl-PC has been proposed to be converted into either arachidonyl-PC in the omega-6 pathway or into EPA-PC, which would be used to be converted into diacylglycerols (DAGs) moieties and then transferred to chloroplasts. In the chloroplasts, the DAGs have been proposed to be galactosylated to the respective monogalactosyldiacylglycerol (MGDG) molecular species. In the last steps, a chloroplastic delta-17 desaturation would be involved in the conversion of ARA-MGDG into galactolipids rich in EPA. Studies conducted with an inhibitor of the delta-6 desaturation involved in the chloroplastic conversion of linoleic acid into α-linolenic acid and using radiolabelled carbon source (acetate) or fatty acid (linoleic acid) permit to elucidate the biosynthesis of EPA. These studies have confirmed the role played by the PC as substrate for the first step in the omega-3 pathway metabolism [282]. The PC were more involved in the desaturation of C18 fatty acids while some species such as PE and betaine lipid (diacylglyceryl trimethylhomoserine DGTS) are preferential substrates for desaturation of C20 fatty acids involved in the EPA synthesis [283]. Moreover, these polar lipids were supposed to be imported to the chloroplastic compartment, from endoplasmic reticulum. The role of EPA and DGTS were suggested to be donors of C20-riched DAG, ARA and EPA, from which these fatty acids were incorporated into the galactolipid classes such as the MGDG [283].

In *Nannochloropsis*, the synthesis of EPA ouside the chloroplast has been proposed by the use of radiolabelled acetate and by kinetic studies in a mutant strain. In these studies, an alteration of the fatty acid composition of lipids was observed, in relation with the EPA synthesis [284]. In the marine green alga *Chlorella minutissima*, the betaine lipid DGTS was characterized by a very high EPA content representing 90% of total fatty acids. By contrast with other algae, the EPA was incorporated into the both *sn*-1 and *sn*-2 positions of the DGTS. Moreover, a close relationship between the DGTS and the PC amounts was observed [285]. In *Chlorella minutissima*, the DGTS level showed a fluctuation which was inversely correlated with the level of MGDG, the other major lipid in this alga. Moreover, the fatty acid composition of the latter was very similar to that of DGTS and, taken together, these findings also support the proposed role of DGTS as a donor of EPA for chloroplast lipids in some microalga species [285]. A mutant of *Porphyridium cruentum*, which was selected on the basis of impaired growth in function of temperatures, has been used to study lipid metabolism [286]. Lipid analysis of the mutant revealed a decreased level of EPA and of the eukaryotic molecular species of MGDG and an increased proportion of TAG by comparison with the wild-type. Pulse-chase labelling of the wild-type algae with radioactive fatty acid precursors showed an initial incorporation of the fatty acids into PC and TAG. In the chase period, the label of these lipids decreased with time while that of chloroplastic lipids, mainly MGDG, continued to increase. In the mutant, however, the labelling of TAG after the pulse was higher than that of the wild-type and decreased only slightly in the subsequent chase. It was concluded that in wild-type *Porphyridium cruentum*, TAG can supply acyl groups for the biosynthesis of eukaryotic species of MGDG [286].

Concerning the DHA synthesis, some microalgae as *Isochrysis galbana* or *Pavlova lutheri* (Haptophyta) are known to produce this fatty acid from C20 metabolism such as EPA [283]. According to previous studies conducted in *Pavlova lutheri*, it could be considered that the DHA synthesis and its incorporation into lipids are specific of the endoplasmic reticulum fraction with a preferential incorporation into the phospholipids and the betaine lipids [265,278]. Furthermore, as EPA is preferentially incorporated into the galactolipid fractions, these lipids being specific of chloroplastidic metabolism, it can be considered that after a chloroplastidic synthesis, EPA would be exported from this compartment into the endoplasmic reticulum in which it would be elongated and desaturated into DHA before being incorporated into specific polar lipids of this compartment.

Another marine microalga, *Crypthecodinium cohnii*, is known to be rich in DHA without accumulation of other PUFAs of the omega-6 and the omega-3 series. In this microalga, the DHA level is about 40%–50% of the total fatty acids without intermediate substrates and products of desaturation and elongation steps. This heterotrophic chloroplast-less microalga has a specific metabolism in which other enzyme pools and subcellular compartments are involved in PUFA synthesis [287].

## 6. Conclusion and Perspectives

Phytoplankton is an exceptionnal source for a variety of bioactive compounds. Among them, pigments and lipids are recognized to have numerous positive effects on health. Very often, synergistic actions between these two kinds of molecules are described. Actually, most studies are done *in vitro*, so it is necessary to develop clinical tests to evaluate their ability, *in vivo*, to avoid some human diseases as well as their possible secondary effects. More, additional intervention trials are required with pigments to precisely determine their absorption, metabolism and potential as natural anti-oxidant, anti-inflammatory and anti-mutagenic compounds *in vivo*.

However, phytoplankton remains largely unexplored and, until now, very few commercial achievements of microalgal biotechnology have emerged [288]. Also, increased harvesting of commercial algae can be considered beneficial for the environment, as they are photosynthetic, *i.e.*, carbon dioxide-absorbing organisms, and thus their cultivation can limit greenhouse gas emissions [289]. Chen *et al.* [290] engineered strategies for simultaneous enhancement of phycocyanin production and CO_2_ fixation with *Spirulina platensis*.

Microalgae are usually cultivated under light. Light is required to get more Chl [291]. However, some species (e.g., *Chlorella protothecoides*, *Crypthecodinium cohnii*, *Schizochytrium limacinum*, *Haematococcus pluvialis*) can be grown heterotrophically [292]. They are cultivated in the dark thanks to a carbon source replacing the more expensive traditional support of light energy. This kind of culture is generally restricted to small volumes of high-value products. Other microorganisms (e.g., *Haematococcus pluvialis*, *Scenedesmus acutus*, *Chlorella vulgaris*, *Nannochloropsis* sp.) also have the ability to grow mixotrophically (with a mix of different sources of carbon and energy). Via this technique, BioReal (Sweden) was the first company to produce and commercialize Asta (15 to 30 T/year) [293].

In this review, we have showed that different parameters can be used to increase pigment and lipid production, e.g., temperature, nutrient starvation, nitrogen concentration, desiccation, CO_2_ level, salt stress or metal stress. Whatever their means of production, some species are cultivated in large volumes and given as food supplement for their global health benefits. This is the case for *Tetraselmis suecica*, *Botryococcus braunii*, *Neochloris oleoabundans*, *Isochrysis* sp., *Chlorella vulgaris*, *Phaeodactylum* *tricornutum* and *Odontella aurita* [110,120]. When particular growth conditions are applied on a microalga species with the goal to optimize the production of one interesting molecule, it is not rare that another interesting molecule is also produced. For instance, Fernandez Sevilla *et al.* [294] succeeded in cultivating a *Scenedesmus almeriensis* strain rich in oils and carotenoids, and in lutein in particular.

Nowadays, production of carotenoids has become one of the most successful activities in algal biotechnology, because of the demand for natural products, instead of being chemically synthesized, despite the fact that synthetic carotenoids have a lower production cost [293]. Carotenoids and especially β-carotene as pigments, are utilised in food and beverages, but also in aquaculture feed [143,148]. Some mg of β-carotene per gramme biomass can now be obtained from microalgae (*Isochrysis*, *Phaeodactylum*, *Chlorella* sp. and *Tetraselmis suecica*) or cyanobacteria (*Spirulina*) mass cultures [110,151]. The estimated market size for natural β-carotene is 10–100 tonnes year^−^^1^ and its price is >750 € kg^−^^1^ [151,295]. Increasing the production of Asta is also an advantageous goal.

The commercial exploitation of the natural microalgal diversity for the production of carotenoids and PUFA has already started up with few strains such as *Chlorella vulgaris* (Trebouxiophyceae), *Dunaliella salina* (Chlorophyceae), *Haematococcus pluvialis* (Chlorophyceae) [249,296] and *Odontella aurita* (Bacillariophyceae) [7,177]. Microalgae are specifically cultivated at an industrial scale for the production of high amounts in EPA and DHA. For example, *Isochrysis* sp. is produced by Innovative Aquaculture Products Ltd. (Lasqueti Island, Canada) and the diatom *Odontella aurita* is produced by BlueBiotech InT (Kollmar, Germany) and Innovalg (Bouin, France). The marine dinoflagellate, *Crypthecodinium cohnii* can produce DHA heterophically. In this way, a DHA-rich oil is produced by the American company Martek Biosciences Corporation [297] and commonly used in supplementation particularly in infant products [186].

Transgenic microalgae can be used as bioreactors for the production of bioactive molecules. This technology is very promising as it would simplify the production process and would lead to a significant decrease of the production costs. To date, a variety of recombinant proteins have been produced experimentally from the nuclear or chloroplast genome of transgenic microalgae, e.g., [298]. For the production of phycoerythrin, Ruiz-Ruiz [299] claims that further improvement could be pursued, e.g., *P. cruentum* biomass production could be optimized in tubular photobioreactors or open-ponds together with molecular biology approaches. For carotenoid production, the use of transgenic microorganisms is still difficult. The knowledge on carotenogenesis pathways in algae is only partial; several genes have been putatively identified in sequenced genomes [222,300]. However, engineering of high-yield algae still exits [301]. Efforts are being developed to engineer fatty acid biosynthesis in microalgae. A recent study shows the importance of a thorough characterization of the algal fatty acid synthase, including protein-protein interactions and regulation, for successful engineering [302]. It is clear from the genome information that most microorganisms have the potential to produce a far greater number of natural products than have been isolated previously [116]. In the future, a better knowledge on pigment and lipid pathways will be determinant to develop new technologies using transgenic marine microorganisms as “high-producers” of bioactive molecules.
